# Comments on: Masters theses from a university medical college: Publication in indexed scientific journals

**DOI:** 10.4103/0301-4738.71698

**Published:** 2010

**Authors:** Suneetha Nithyanandam, Usha Vasu

**Affiliations:** Department of Ophthalmology, St. John’s Medical College, Bangalore - 560 034, India

Dear Editor,

Congratulations to Dhaliwal *et al*., for their paper titled ’Masters theses from a University medical college – Publication in indexed scientific journals’.[1] It is thought-provoking and a ‘must read’ for academicians. Congratulations to the editorial team also for publishing articles of this genre.[2] We share some of our own thoughts here.

The publication rate of 30% in this report is very good. However, the number of publications from the institution could have additionally been ascertained from the respective departments, as the departments would best know their publications. Additionally, the authors could have evaluated the effect of clinical specialties versus non-clinical specialties on the publication rate. There is a perception among medical college faculty that the publications tend to be higher in non-clinical subjects.

Knowledgeable guides are vital to thesis development. Rehashing earlier topics also results in unworthiness for publication, but we often find several successive theses on the same topic - one of us (SN) recently evaluated five Masters’ theses. Four of these were related to cataract; three compared outcomes and complications in phacoemulsification, manual small incision surgery and standard extra-capsular surgeries in various permutations and combinations! Someone was reinventing the wheel! The topics should have been rejected in the synopsis stage. A mechanism for checking repetition of topics is imperative for all universities. We could also add poor funding, absence of good diagnostics, lack of follow-up and poor documentation as other causes – but there are many solutions available to these problems.

For guides and residents alike, there are many advantages of choosing potentially publishable topics. Publications enhance the reputation of the department and the institution. They are Medical Council of India (MCI) requirements for promotions.[3] Those planning further training can buttress their curriculum vitae with good publications. Publication should become the focus of thesis planning – use of a good “research question” and well-structured theses will then follow. As students are barely three months into their training when synopses are submitted, guides and co-guides should assist them in developing thesis proposals.

Some good theses do not get published as they are never sent for publication; the resident after course completion is busy finding suitable employment or further career openings, and has no time to think of publication. Here again, the guides could lend a helping hand in facilitating submission for publication, lest the knowledge gained by the research work remains in the archives of the institutional library.
